# LncRNA GAS5 Competitively Combined With miR-21 Regulates PTEN and Influences EMT of Peritoneal Mesothelial Cells via Wnt/β-Catenin Signaling Pathway

**DOI:** 10.3389/fphys.2021.654951

**Published:** 2021-08-30

**Authors:** Yi Fan, Xingxu Zhao, Jianfei Ma, Lina Yang

**Affiliations:** ^1^Department of Nephrology, The First Affiliated Hospital of China Medical University, Shenyang, China; ^2^Department of Geriatrics, The First Affiliated Hospital of China Medical University, Shenyang, China

**Keywords:** lncRNA GAS5, miR-21, PTEN, Wnt/β-catenin, EMT, HPMCs

## Abstract

**Objective:**

Epithelial-mesenchymal transition (EMT) is an important factor leading to peritoneal fibrosis (PF) in end-stage renal disease (ESRD) patients. The current research aimed to evaluate the effect of long non-coding RNA growth arrest-specific 5 (lncRNA GAS5) in human peritoneal mesothelial cells (HPMCs) EMT and explore the potential molecular mechanisms.

**Materials and Methods:**

HPMCs were cultured under control conditions or with high glucose (HG). The cells were then treated with lncRNA GAS5, lncRNA GAS5 siRNA, with or without miR-21 inhibitor and PTEN transfection. Expression of lncRNA GAS5, miR-21, α-SMA, Vimentin, E-cadherin, phosphatase and tensin homolog deleted on chromosome ten (PTEN), Wnt3a, and β-catenin were measured by real time PCR and Western blotting. Bioinformatics analyses were used to test the specific binding sites between the 3′ UTR of the PTEN gene, miR-21, and lncRNA GAS5. Rescue experiments were performed to confirm the lncRNA GAS5/miR-21/PTEN axis in HPMC EMT.

**Results:**

We found that HG-induced EMT decreased lncRNA GAS5 and that overexpression of lncRNA GAS5 can attenuate EMT in HPMCs. In addition, lncRNA GAS5 regulated HG-induced EMT through miR-21/PTEN. Cotransfection of miR-21 inhibitors remarkably increased PTEN expression and attenuated EMT in lncRNA GAS5 knockdown HPMCs. Moreover, rescue experiments showed that overexpression of PTEN attenuated the EMT effects of lncRNA GAS5 siRNA in HPMCs. We also confirmed that the Wnt/β-catenin pathway was stimulated in lncRNA GAS5/miR-21/PTEN-mediated EMT.

**Conclusion:**

Our research showed that lncRNA GAS5 competitively combined with miR-21 to regulate PTEN expression and influence EMT of HPMCs via the Wnt/β-catenin signaling pathway. This study provides novel evidence that lncRNA GAS5 may be a potential therapeutic target for HPMC EMT.

## Introduction

Peritoneal dialysis (PD) is one of the important alternative therapies for end-stage renal disease (ESRD), and it has been more widely used recently. However, long-term PD exposes peritoneal mesothelial cells to biologically incompatible PD fluid, which leads to loss of ultrafiltration and is an important reason for PD patient withdrawal from PD treatment (Zhang et al., 2012). Recently, researchers have found that epithelial-mesenchymal transition (EMT), the initial reversible step in the peritoneal fibrosis (PF) process, is an important factor leading to PF in patients with PD. Therefore, exploring the mechanisms of EMT and taking measures to effectively delay or even reverse its progress could prolong the dialysis period for PD patients and improve quality of life for ESRD patients.

The latest research suggests that differentially expressed long non-coding RNAs (lncRNAs) may play a vital regulatory role in the occurrence and development of organ fibrosis (Cao et al., 2013). One study found that 232 lncRNAs were differentially expressed in the PF mouse model used, indicating that lncRNAs are also involved in the regulation of PF (Liu et al., 2015). Among these lncRNAs, lncRNA GAS5 has been shown to regulate organ fibrosis in liver (Gong et al., 2018), heart (Liu H. L. et al., 2019), and kidney (Zhang et al., 2020) through binding of competing endogenous RNAs (ceRNAs) to microRNAs (miRs) and by directly binding proteins. miR-21 has also been shown to induce organ fibrosis in liver (Noetel et al., 2012), lungs (Liu et al., 2010), heart (Brønnum et al., 2013), and kidneys (Glowacki et al., 2013). Researchers showed that lncRNA GAS5 can target the miR-21 gene and regulate cell proliferation and apoptosis (Liu et al., 2018; Liu K. et al., 2019). Our previous research demonstrated that miR-21 targeting of phosphatase and tensin homolog deleted on chromosome ten (PTEN) played an important role in the HG-induced EMT of human peritoneal mesothelial cells (HPMCs) (Yang et al., 2018).

The Wnt signaling pathway is known to regulate organ fibrosis and EMT (Akcora et al., 2018; Wang et al., 2018). The Wnt/β-catenin signaling pathway has been confirmed to be involved in peritoneal EMT, specifically (Yang et al., 2017).

Based on the above findings, our research aimed to investigate the role of lncRNA GAS5 in the miR-21/PTEN axis and the effect of Wnt/β-catenin signaling pathway in EMT of HPMCs.

## Materials and Methods

### Cell Culture and Treatments

HPMCs and HMrSV5 were cultured in RPMI 1640 medium containing 10% fetal bovine serum (FBS) at 37°C in 5% CO_2_. After adherence to the wall, the cells were digested and passaged at ratios of 1:3 to 1:4. Following our previously published methods (Yang et al., 2018), HPMCs were cultured with 5.5 mmol/L glucose (normal glucose, Control), 2.5% HG (126 mmol/L) and mannitol as the high osmotic pressure group (5.5 mM glucose + 120.5 mmol/L mannitol)for 24 h, then cells were collected for subsequent experiments. lncRNA GAS5 overexpression plasmids and lncRNA GAS5 siRNA were purchased from GenePharma (Shanghai, China). The empty pcDNA3.1 vector was used as a control. miR-21 inhibitors and respective negative control miRs (NC-miR) were bought from RiboBio (Guangzhou, China). HPMCs were transfected with plasmids or oligonucleotides using Lipofectamine 3000 (Invitrogen) according to the manufacturer’s instructions. After stimulation with 2.5% HG for 24 h, cells were then collected for real time PCR and western blotting. Transfection efficacy was verified by quantitative real-time polymerase chain reaction (RT-qPCR) analysis 24 h after transfection.

### Western Blotting

Western blot analysis was conducted as previously published (Yang et al., 2018). Total protein was extracted from lysed cells using RIPA buffer (Beyotime, Shanghai, China) and then quantified using the Pierce BCA Protein Assay Kit (Thermo Fisher Scientific, Rockford, IL). About 50 μg protein from HPMCs were subjected to 10% SDS-PAGE at 70 V for 2 h before being transferred onto a PVDF membrane (Millipore, Billerica, MA) at 100 V for 1 h. After blocking with 5% non-fat dry milk, the membranes were incubated overnight at 4°C with 1:1,000 dilutions for primary antibodies and 1:5,000 for secondary antibodies. Images of protein bands were captured by UVP (G-BOX EF) after development with ECL reagents (Pierce Biotechnology, Inc.). Quantitation of protein levels were measured by densitometry from three independent experiments, normalized to loading control β-actin.

### Real-Time PCR

Total RNA was extracted from HPMCs using Trizol reagent (Invitrogen) according to the manufacturer’s instruction. First-strand cDNAs were synthesized using a Reverse Transcription System kit according to the manufacturer’s protocol (Takara). Real-time quantitative PCR was conducted by the SYBR Premix Ex Taq II kit (Takara) with the ABI 7500 Real-Time PCR System (Applied Biosystems, CA). The relative gene expression levels were measured using the 2^–ΔΔC^*^*t*^* method after normalization with GAPDH or U6 (RiboBio, Guangzhou, China). Experiments were repeated at least three times. The primer sequences (RiboBio, Guangzhou, China) were as follows: lncRNA GAS5 forward primer: 5′-CAGATGCAGTGTGGCTCTGGA-3′ and reverse primer: 5′-TGTGTGCCAATGGCTTGAGTTAG-3′; miR-21-5p Reverse transcription (RT) primer: 5′-GTCGTATCCAGTGCAGG GTCCGAGGTATTCGCACTGGATACGACTCAACATCAGT-3′, forward primer: 5′-GGCGGTAGCTTATCAGACTGATG-3′, and reverse primer: 5′-GTGCAGGGTCCGAGGTATTC-3′; U6 RT primer: 5′-AACGCTTCACGAATTTGCGT-3′, forward primer: 5′-CTCGCTTCGGCAGCACA-3′, and reverse primer: 5′-AACGCTTCACGAATTTGCGT-3′; and GAPDH forward primer: 5′-GCACCGTCAAGGCTGAGAAC-3′ and reverse primer: 5′-TGGTGAACACGCCAGTGGA-3′.

### Immunofluorescence Staining With β-Catenin

The cell slides were placed in 24-well plate, HPMCs were seeded at 1 × 10^5^ cells/well, and cultured overnight in a 37°C, 5% CO2 incubator. After 4% paraformaldehyde fixation, 0.25% Triton X-100 permeabilization, 5% BSA (Sigma-Aldrich; Merck Millipore) blocking and PBS washing, 10% BSA diluted β-catenin primary antibody was incubated overnight at 4°C; after PBS washing for 5 min × 3 times, 10% BSA diluted secondary antibody and DAPI were incubated at 37°C for 1 h. Stained cells were visualized using a fluorescence microscope (Nikon ECLIPSE Ti; Nikon Corporation, Tokyo, Japan).

### Statistical Analyses

All statistical analyses were carried out using SPSS (version 18) software. Quantitative data were presented as mean ± SEM. Student *t* tests were carried out for comparisons between two groups and standard ANOVA methodology was carried out for comparisons among multiple groups (*P* < 0.05 was considered statistically significant).

## Results

### HG-Induced EMT Decreased IncRNA GAS5 While IncRNA GAS5 Overexpression Attenuated EMT in HPMCs

**FIGURE 1:**
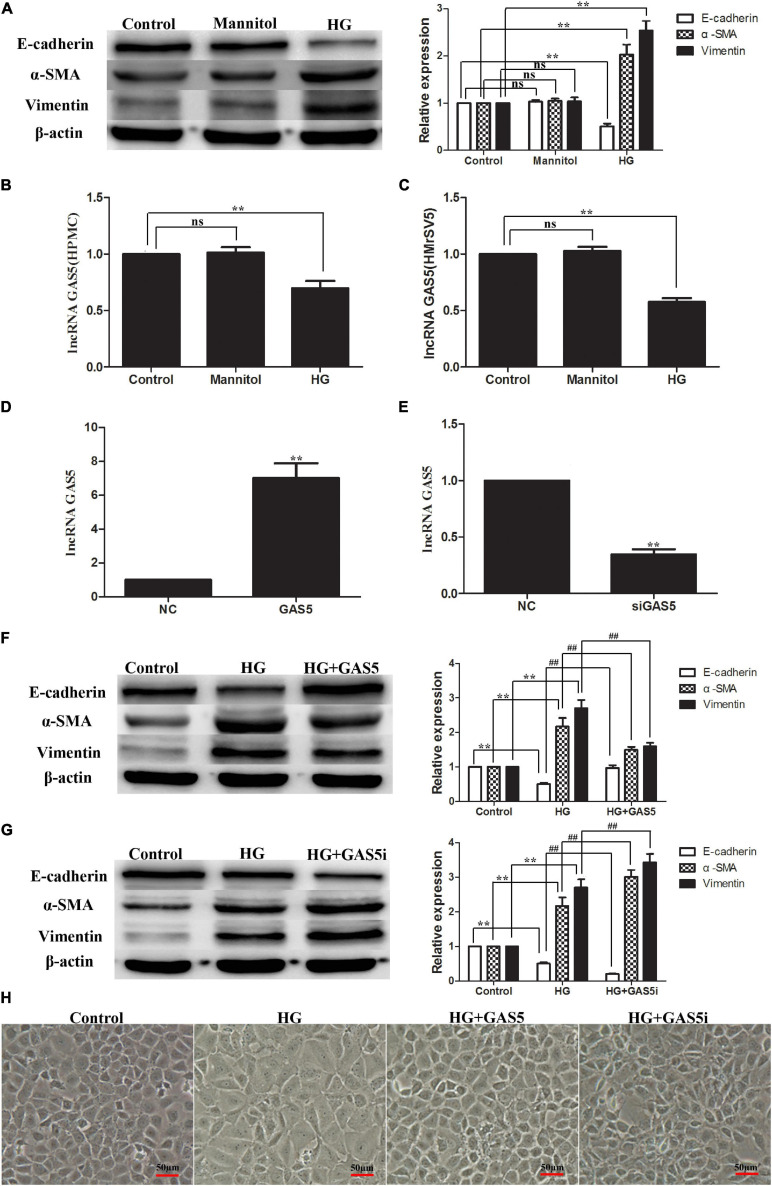
HG-induced EMT decreased lncRNA GAS5 while lncRNA GAS5 overexpression attenuated EMT in HPMCs. HPMCs were cultured with 2.5% HG and mannitol for 24 h. **(A)** HG treatment induced EMT in HPMCs. **(B,C)** HG decreased lncRNA GAS5 expression significantly in HPMCs and HMrSV5. **(D,E)** HPMCs were transfected with pcDNA3.1-GAS5 and lncRNA GAS5 siRNA. **(F)** Compared with HG group, lncRNA GAS5 attenuated the progress of the HPMCs EMT. **(G)** Compared with HG group, lncRNA GAS5 siRNA enhanced the HPMCs EMT. **(H)** Normal cells showed a paving stone-like appearance, HPMCs were changed to a fibroblast-like morphology after incubation with HG, lncRNA GAS5 reversed the morphological alteration induced by HG, while lncRNA GAS5 siRNA exacerbated the morphological alteration. All the results are represented as mean ± SEM from three independent experiments. (**P < 0.01 vs. control, ^##^*P* < 0.01 vs. HG).

Compared with the control group, stimulation of HPMCs with 2.5% HG for 24 h increased the expression of α-SMA and Vimentin significantly while it decreased the expression of E-cadherin, which means that HG treatment induced EMT in HPMCs (Figure 1A). HG treatment also significantly decreased lncRNA GAS5 expression in HPMCs and HMrSV5 cell lines (Figures 1B,C). In order to test whether lncRNA GAS5 could modulate HG-induced EMT, HPMCs were transfected with pcDNA3.1-GAS5 and GAS5 siRNA. The expression of lncRNA GAS5 was significantly upregulated and downregulated, respectively (Figures 1D,E). Compared with HG group, overexpression of lncRNA GAS5 significantly decreased the levels of α-SMA and Vimentin while increasing the level of E-cadherin (Figure 1F), which suggests that lncRNA GAS5 attenuated EMT progress of the HPMCs. Moreover, lncRNA GAS5 siRNA exacerbated the HPMCs’ EMT (Figure 1G). The cell morphology alterations of the above groups were also observed. Normal cells showed a paving stone-like appearance, which was changed to a fibroblast-like morphology after incubation with HG, overexpression of lncRNA GAS5 reversed the changes in cell morphology induced by HG, while lncRNA GAS5 siRNA exacerbated the morphological alteration (Figure 1H). These data demonstrate that lncRNA GAS5 modulated HG-induced EMT in HPMCs.

### LncRNA GAS5 Regulated HG-Induced EMT Through miR-21/PTEN

**FIGURE 2:**
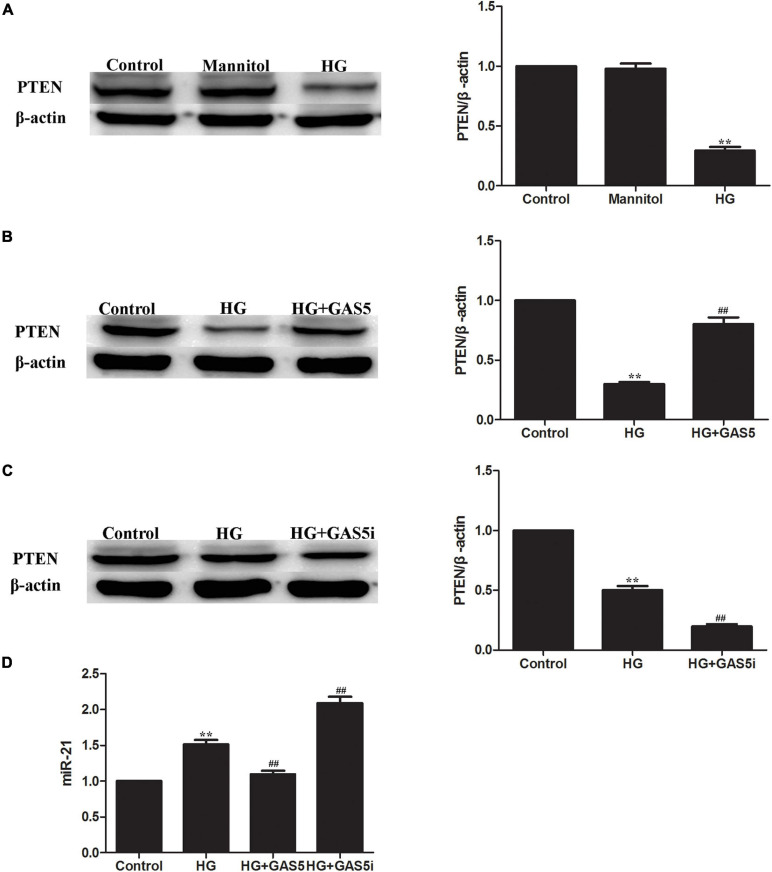
LncRNA GAS5 regulated HG-induced EMT through miR-21/PTEN. **(A)** Western blotting showed that PTEN was downregulated in HG-induced EMT. **(B)** Compared with HG group, overexpression of lncRNA GAS5 upregulated PTEN expression. **(C)** Compared with HG group, lncRNA GAS5 siRNA downregulated PTEN expression. **(D)** Real-time PCR showed that miR-21 was upregulated in HG-induced EMT, compared with HG group, overexpression of lncRNA GAS5 downregulated miR-21 and lncRNA GAS5 siRNA upregulated miR-21. Each value represents the mean ± SEM (*n* = 3) (^∗∗^*P* < 0.01 vs. control, ^##^*P* < 0.01 vs. HG).

To confirm whether lncRNA GAS5 regulate HG-induced EMT through miR-21/PTEN, the expression of miR-21 and PTEN were measured after pcDNA3.1-GAS5 and GAS5 siRNA transfection in HPMCs. Western blotting showed that PTEN was downregulated in HG-induced EMT (Figure 2A). Compared with HG group, overexpression of lncRNA GAS5 upregulated PTEN expression (Figure 2B); in contrast, lncRNA GAS5 siRNA downregulated PTEN expression (Figure 2C). Meanwhile, Real-time PCR showed that miR-21 was upregulated in HG-induced EMT, overexpression of lncRNA GAS5 downregulated miR-21 and lncRNA GAS5 siRNA upregulated miR-21 when compared with HG group (Figure 2D). These data demonstrate that lncRNA GAS5 regulated HG-induced EMT through miR-21/PTEN.

### IncRNA GAS5 Regulated PTEN by Competitively Binding to miR-21

**FIGURE 3:**
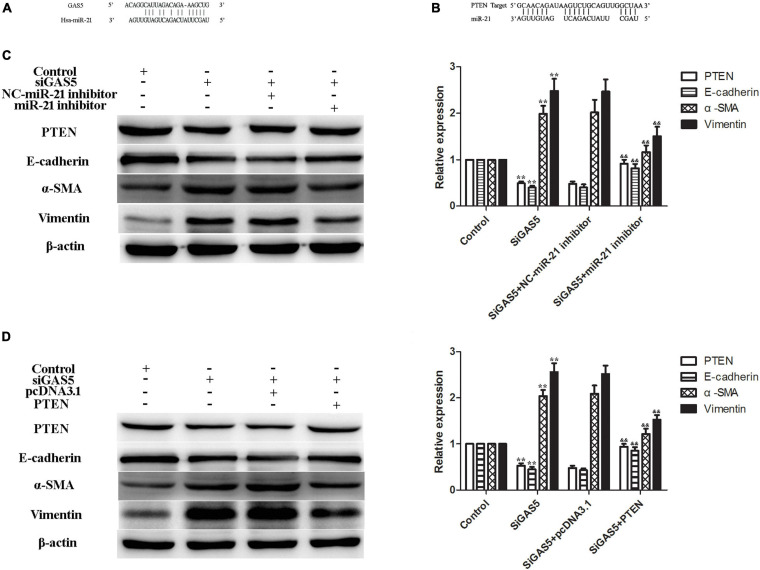
LncRNA GAS5 regulated PTEN by competitively binding to miR-21. **(A,B)** Bioinformatics analyses showed that there were specific binding sites between lncRNA GAS5 and sequences of miR-21 **(A)** and between the 3′ UTR of the PTEN gene and sequences of miR-21 (B). **(C)** miR-21 inhibitor increased PTEN and attenuated lncRNA GAS5 siRNA-induced EMT of HPMCs. **(D)** PTEN reversed the lncRNA GAS5 siRNA-induced EMT of HPMCs. Each value represents the mean ± SEM (*n* = 3) (**P < 0.01 vs. control, ^&⁣&^*P* < 0.01 vs. siGAS5).

In a previous study, we reported that miR-21 targeted PTEN during EMT of HPMCs (Yang et al., 2018). In this study, we further verified that lncRNA GAS5 regulated PTEN by competitively binding to miR-21 in HPMCs. Bioinformatics analyses showed that there were specific binding sites between the 3′ UTR of the PTEN gene and sequences of miR-21 and between lncRNA GAS5 and sequences of miR-21 (Figures 3A,B).

To further confirm the regulatory mechanism of the lncRNA GAS5/miR-21/PTEN axis in HPMC EMT, two rescue experiments were performed. The results showed that the expression of PTEN was downregulated and EMT was enhanced after the lncRNA GAS5 siRNA transfection; additionally, the expression of PTEN was increased and EMT was attenuated following treatment of HPMCs with a miR-21 inhibitor, the expression of PTEN and EMT markers showed no difference between GAS5 siRNA and GAS5 siRNA + NC miR-21 inhibitor groups (Figure 3C). In the next experiment, HPMCs were transfected with lncRNA GAS5 siRNA and PTEN plasmid individually and in combination. Transfection of lncRNA GAS5 siRNA alone reduced the gene expression of PTEN and enhanced EMT progress of the HPMCs. When the cells were simultaneously transfected with lncRNA GAS5 siRNA and PTEN, EMT progress was significantly reversed (Figure 3D). These data demonstrate that lncRNA GAS5 regulated PTEN by competitively binding to miR-21.

### Wnt/β-Catenin Pathway Was Stimulated in IncRNA GAS5/miR-21/PTEN-Mediated EMT

**FIGURE 4:**
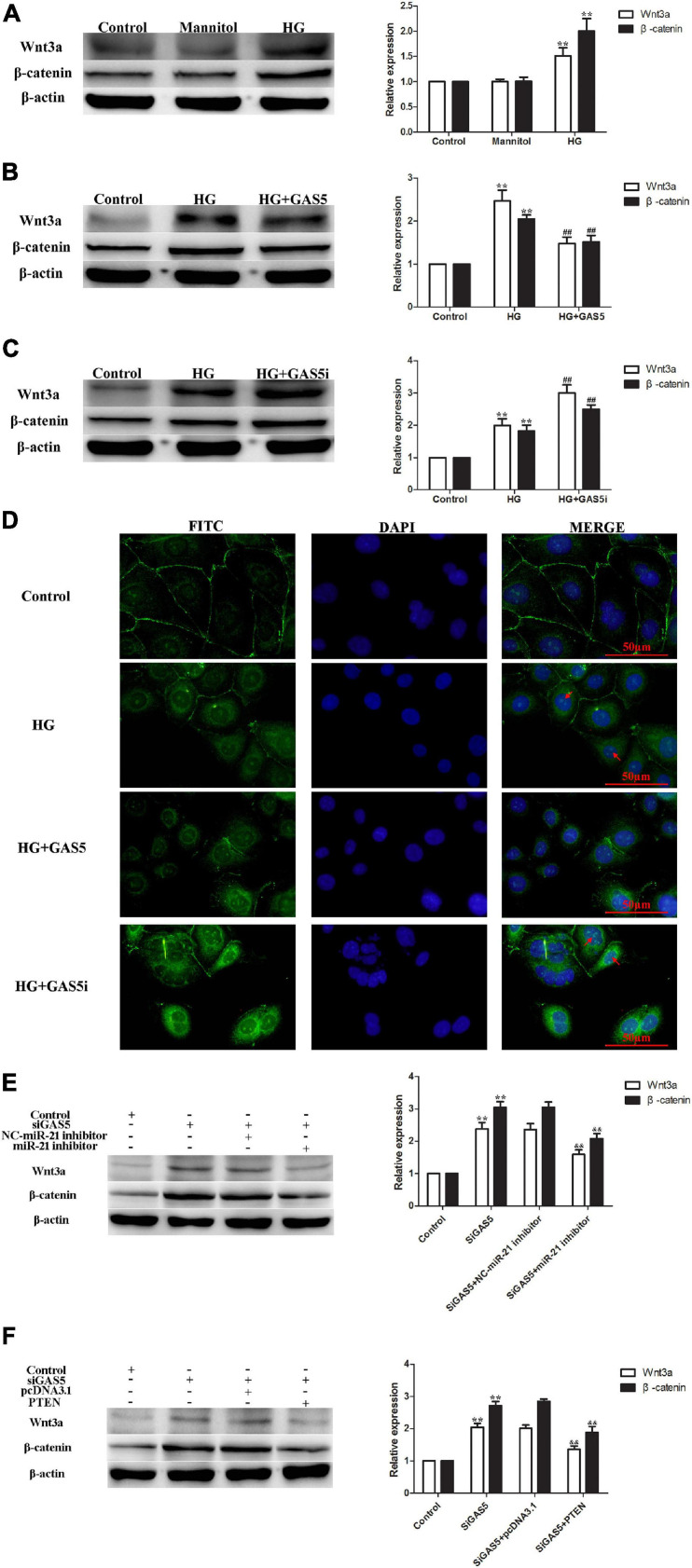
The Wnt/β-catenin pathway was stimulated in lncRNA GAS5/miR-21/PTEN-mediated EMT. **(A)** HG stimulated the Wnt/β-catenin pathway. **(B)** lncRNA GAS5 overexpression significantly downregulated the Wnt/β-catenin pathway. **(C)** lncRNA GAS5 siRNA significantly upregulated the Wnt/β-catenin pathway. **(D)** Immunofluorescence staining demonstrated that an increase of nuclear β-catenin accumulation was evident in HG-induced HPMCs EMT, lncRNA GAS5 overexpression attenuated β-catenin nuclear localization and lncRNA GAS5 siRNA aggravated nuclear localization. The arrows indicate nuclear localization of β-catenin. **(E)** miR-21 inhibitor attenuated lncRNA GAS5 siRNA stimulated Wnt/β-catenin pathway. **(F)** PTEN reversed lncRNA GAS5 siRNA stimulated Wnt/β-catenin pathway. Each value represents the mean ± SEM (*n* = 3) (**P < 0.01 vs. control, ^##^*P* < 0.01 vs. HG, ^&⁣&^*P* < 0.01 vs. siGAS5).

HG stimulation of HPMCs significantly increased cellular expression of Wnt3a and β-catenin (Figure 4A). Compared with HG group, lncRNA GAS5 overexpression decreased the levels of Wnt3a and β-catenin (Figure 4B), while lncRNA GAS5 siRNA significantly increased the levels of Wnt3a and β-catenin (Figure 4C). In addition, immunofluorescence staining demonstrated that an increase of nuclear β-catenin accumulation was evident in HG-induced HPMCs EMT, lncRNA GAS5 overexpression attenuated β-catenin nuclear localization and lncRNA GAS5 siRNA aggravated nuclear localization (Figure 4D). These data demonstrate that the Wnt/β-catenin pathway was involved in lncRNA GAS5-mediated EMT in HPMCs.

To confirm that the Wnt/β-catenin pathway was stimulated in lncRNA GAS5/miR-21/PTEN-mediated EMT, rescue experiments were performed. The levels of Wnt3a and β-catenin were increased after lncRNA GAS5 siRNA transfection, but were decreased following miR-21 inhibitor treatment, the expression of Wnt3a and β-catenin showed no difference between GAS5 siRNA and GAS5 siRNA + NC miR-21 inhibitor groups (Figure 4E). Additionally, when HPMCs were transfected with lncRNA GAS5 siRNA and PTEN plasmid together, the increased expression of Wnt3a and β-catenin was significantly reversed (Figure 4F). These data demonstrate that wnt/β-catenin pathway was stimulated in lncRNA GAS5/miR-21/PTEN-mediated EMT.

## Discussion

Compared with traditional hemodialysis, PD can better protect residual renal function, maintain a stable internal environment, reduce the negative impact on the cardiovascular system, and improve the prognosis of ESRD patients (Yáñez-Mó et al., 2003). However, the low pH, high glucose, lactate, and glucose degradation products of PD fluid cause chronic inflammation and injury of HPMCs, eventually causing PF. Therefore, it is scientifically and clinically valuable to explore the mechanism of PF and find effective prevention and treatment measures. High glucose PD fluid can cause peritoneal mesothelial cells to lose their cellular characteristics such as loss of cell polarity and adhesion, reduced expression of E-cadherin, transformation into fibroblasts, enhanced migration and invasion ability, and overexpression of α-SMA and Vimentin, which includes EMT of HPMCs (Guo et al., 2018). As the initial and reversible step in the PF process, EMT occurs in the early stages of PF and plays a key role in PF (de Graaff et al., 2010).

Previous studies have shown that miR-21 targeting of PTEN is important in the process of organ fibrosis (Zhou et al., 2017; Zhang and Cui, 2018). The regulation of the miRNA-competing endogenous RNA (ceRNA) network has become a research focal point in recent years, including the regulation of miR-21 specifically which plays a key role in peritoneal EMT (Yang et al., 2018). Furthermore, the regulation of miRNAs by ceRNA is currently receiving much attention. The lncRNA located in the cytoplasm can function like ceRNA by competitively binding to miRNA, absorbing it like a sponge, and blocking the miRNA’s silencing of target mRNA (Fatica and Bozzoni, 2014). The latest research suggests that lncRNAs have a specific expression profile in fibrotic tissues such as lung, kidney, liver, heart, and peritoneum (Cao et al., 2013; Liu et al., 2015). Several researchers revealed that lncRNA-AV310809, 6030408B16RIK, and AK089579 modulate peritoneal EMT (Wei et al., 2019; Zhang et al., 2019; Wang Z. et al., 2020). However, the specific role and related mechanisms of lncRNA in peritoneal fibrosis remain unclear.

Our current study showed that lncRNA GAS5 attenuated EMT of HPMCs. Further experiments showed that lncRNA GAS5 regulated PTEN by competitively binding to miR-21, which played an important role in the EMT process of HPMCs. The association between lncRNA GAS5 and miR-21 has been reported in many physiological and pathological processes, including cardiac fibrosis (Tao et al., 2017), osteoarthritis (Song et al., 2014), and cancer (Wang C. et al., 2020). For the first time, we confirmed the interaction between lncRNA GAS5 and miR-21 in HPMCs, as miR-21 inhibitor treatment and overexpression of PTEN in HPMCs blocked the effects of GAS5 siRNA on EMT. Our study also confirmed that the miR-21/PTEN axis might be partly involved in lncRNA GAS5-mediated EMT of HPMCs.

Recent studies have found that the Wnt signaling pathway plays a vital role in the process of organ fibrosis and EMT; indeed, the Wnt/β-catenin signaling pathway has been confirmed to participate in the occurrence of peritoneal EMT (Yang et al., 2017; Akcora et al., 2018; Wang et al., 2018). Studies have found that PTEN can regulate EMT by reducing Wnt expression (Zhao et al., 2018), suggesting that PTEN may interact with Wnt to regulate the progress of peritoneal EMT. Our results confirmed that the Wnt/β-catenin pathway was stimulated in lncRNA GAS5/miR-21/PTEN-mediated EMT.

**FIGURE 5:**
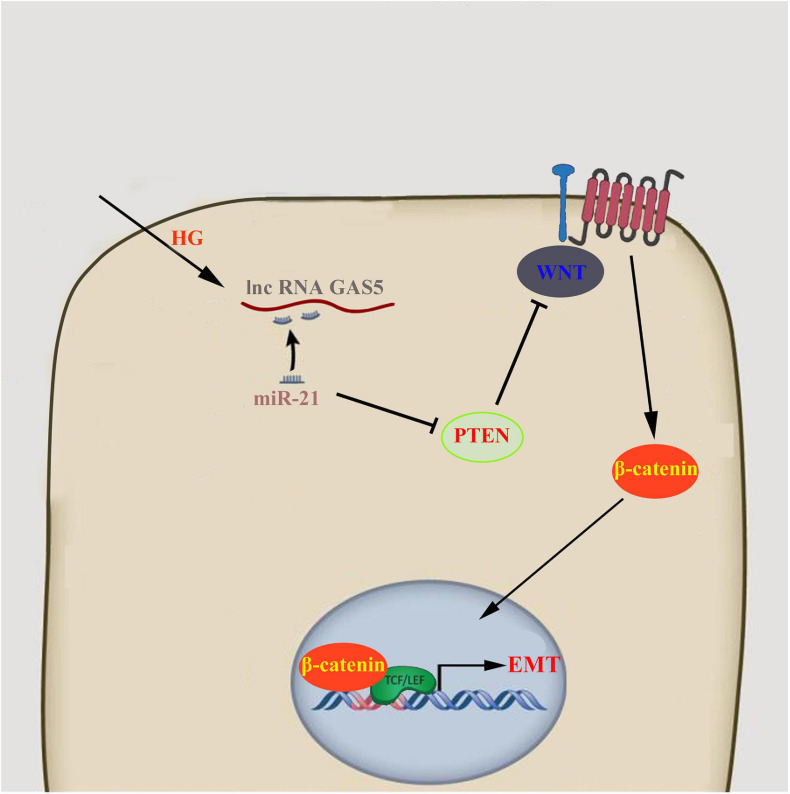
lncRNA GAS5/miR-21/PTEN regulated HPMCs EMT via Wnt/β-catenin pathway.

In conclusion, our study showed that high glucose caused the reduction of lncRNA GAS5, which regulated PTEN through competitively binding to miR-21, stimulated Wnt/β-catenin pathway, and eventually lead to the occurrence of EMT in HPMCs (Figure 5). The results of this study have important implications for clarifying the mechanism of peritoneal EMT and for exploring new predictive markers and therapeutic targets.

## Data Availability Statement

The raw data supporting the conclusions of this article will be made available by the authors, without undue reservation.

## Author Contributions

LY and YF mainly performed experiments, interpreting data, and drafting the manuscript. XZ participated in the interpretation of data and revised the manuscript. JM designed, supervised this study, and revised the manuscript. All authors read and approved the final manuscript.

## Conflict of Interest

The authors declare that the research was conducted in the absence of any commercial or financial relationships that could be construed as a potential conflict of interest.

## Publisher’s Note

All claims expressed in this article are solely those of the authors and do not necessarily represent those of their affiliated organizations, or those of the publisher, the editors and the reviewers. Any product that may be evaluated in this article, or claim that may be made by its manufacturer, is not guaranteed or endorsed by the publisher.

## References

[B1] Akcora B. Ö Storm G. Bansal R. (2018). Inhibition of canonical WNT signaling pathway by β-catenin/CBP inhibitor ICG-001 ameliorates liver fibrosis in vivo through suppression of stromal CXCL12. *Biochim. Biophys. Acta* 1864 804–818. 10.1016/j.bbadis.2017.12.001 29217140

[B2] Brønnum H. Andersen D. C. Schneider M. Sandberg M. B. Eskildsen T. Nielsen S. B. (2013). MiR-21 promotes fibrogenic epithelial-to-mesenchymal transition of epicardial mesothelial cells involving programmed cell death 4 and Sprouty-1. *PLoS One* 8:e56280. 10.1371/journal.pone.0056280 23441172 PMC3575372

[B3] Cao G. Zhang J. Wang M. Song X. Liu W. Mao C. (2013). Differential expression of long non-coding RNAs in bleomycin-induced lung fibrosis. *Int. J. Mol. Med.* 32 355–364. 10.3892/ijmm.2013.1404 23732278

[B4] de Graaff M. Zegwaard A. H. Zweers M. M. Vlijm A. de Waart D. R. Vandemaele F. (2010). The effects of a dialysis solution with a combination of glycerol/amino acids/dextrose on the peritoneal membrane in chronic renal failure. *Perit. Dial. Int.* 30 192–200. 10.3747/pdi.2008.00159 20124192

[B5] Fatica A. Bozzoni l (2014). Long non-coding RNAs: new players in cell differentiation and development. *Nat. Rev. Genet.* 15 7–21. 10.1038/nrg3606 24296535

[B6] Glowacki F. Savary G. Gnemmi V. Buob D. Van der Hauwaert C. Lo-Guidice J. M. (2013). Increased circulating miR-21 levels are associated with kidney fibrosis. *PLoS One* 8:e58014. 10.1371/journal.pone.0058014 23469132 PMC3585177

[B7] Gong Z. Deng C. Xiao H. Peng Y. Hu G. Xiang T. (2018). Effect of Dahuang Zhechong pills on long non-coding RNA growth arrest specific 5 in rat models of hepatic fibrosis. *J. Tradit. Chin. Med.* 38 190–196. 10.1016/j.jtcm.2018.04.007 32186058

[B8] Guo R. Hao G. Bao Y. Xiao J. Zhan X. Shi X. (2018). MiR-200a negatively regulates TGF-β1 induced epithelial-mesenchymal transition of peritoneal mesothelial cells by targeting ZEB1/2 expression. *Am. J. Physiol. Renal Physiol.* 314 F1087–F1095. 29357421 10.1152/ajprenal.00566.2016

[B9] Liu G. Friggeri A. Yang Y. Milosevic J. Ding Q. Thannickal V. J. (2010). miR-21 mediates fibrogenic activation of pulmonary fibroblasts and lung fibrosis. *J. Exp. Med.* 207 1589–1597. 10.1084/jem.20100035 20643828 PMC2916139

[B10] Liu H. L. Chen C. H. Sun Y. J. (2019). Overexpression of lncRNA GAS5 attenuates cardiac fibrosis through regulating PTEN/MMP-2 signal pathway in mice. *Eur. Rev. Med. Pharmacol. Sci.* 23 4414–4418. 31173316 10.26355/eurrev_201905_17949

[B11] Liu K. Liu C. Zhang Z. (2019). lncRNA GAS5 acts as a ceRNA for miR-21 in suppressing PDGF-bb-induced proliferation and migration in vascular smooth muscle cells. *J. Cell. Biochem.* 120 15233–15240. 10.1002/jcb.28789 31069831

[B12] Liu X. She Y. Wu H. Zhong D. Zhang J. (2018). Long non-coding RNA Gas5 regulates proliferation and apoptosis in HCS-2/8 cells and growth plate chondrocytes by controlling FGF1 expression via miR-21 regulation. *J. Biomed. Sci.* 25:18. 29490650 10.1186/s12929-018-0424-6 PMC5830091

[B13] Liu Y. Guo R. Hao G. Xiao J. Bao Y. Zhou J. (2015). The expression profiling and ontology analysis of non-coding RNAs in peritoneal fibrosis induced by peritoneal dialysis fluid. *Gene* 564 210–219. 10.1016/j.gene.2015.03.050 25827714

[B14] Noetel A. Kwiecinski M. Elfimova N. Huang J. Odenthal M. (2012). MicroRNA are central players in anti- and profibrotic gene regulation during liver fibrosis. *Front. Physiol.* 3:49. 10.3389/fphys.2012.00049 22457651 PMC3307137

[B15] Song J. Ahn C. Chun C. H. Jin E. J. (2014). A long non-coding RNA, GAS5, plays a critical role in the regulation of miR-21 during osteoarthritis. *J. Orthop. Res.* 32 1628–1635. 25196583 10.1002/jor.22718

[B16] Tao H. Zhang J. G. Qin R. H. Dai C. Shi P. Yang J. J. (2017). LncRNA GAS5 controlscardiac fibroblast activation and fibrosis by targeting miR-21via PTEN/MMP-2 signaling pathway. *Toxicology* 386 11–18. 10.1016/j.tox.2017.05.007 28526319

[B17] Wang C. Ke S. Li M. Lin C. Liu X. Pan Q. (2020). Downregulation of LncRNA GAS5 promotes liver cancer proliferation and drug resistance by decreasing PTEN expression. *Mol. Genet. Genomics* 295 251–260. 10.1007/s00438-019-01620-5 31705194

[B18] Wang Y. Zhou C. J. Liu Y. (2018). Wnt signaling in kidney development and disease. *Prog. Mol. Biol. Transl. Sci.* 153 181–207. 10.1016/bs.pmbts.2017.11.019 29389516 PMC6008255

[B19] Wang Z. Zhou Z. Ji W. Sun L. Man Y. Wang J. (2020). Silencing of lncRNA 6030408B16RIK prevents ultrafiltration failure in peritoneal dialysis via microRNA-326-3p-mediated WISP2 downregulation. *Biochem. J.* 477 1907–1921. 10.1042/BCJ20190877 32255479

[B20] Wei X. Huang H. Bao Y. Zhan X. Zhang L. Guo R. (2019). Novel long non-coding RNA AV310809 promotes TGF-β1 induced epithelial-mesenchymal transition of human peritoneal mesothelial cells via activation of the Wnt2/β-catenin signaling pathway. *Biochem. Biophys. Res. Commun.* 513 119–126. 10.1016/j.bbrc.2019.03.071 30935692

[B21] Yáñez-Mó M. Lara-Pezzi E. Selgas R. Ramírez-Huesca M. Domínguez-Jiménez C. Jiménez-Heffernan J. A. (2003). Peritoneal dialysis and epithelial-to-mesenchymal transition of mesothelial cells. *N. Engl. J. Med.* 348 403–413. 12556543 10.1056/NEJMoa020809

[B22] Yang D. Fu W. Li L. Xia X. Liao Q. Yue R. (2017). Therapeutic effect of a novel Wnt pathway inhibitor on cardiac regeneration after myocardial infarction. *Clin. Sci. (Lond.)* 131 2919–2932. 10.1042/cs20171256 29162747

[B23] Yang L. Fan Y. Zhang X. Gao L. Ma J. (2018). Role of miRNA-21/PTEN on the high glucose-induced EMT in human mesothelial peritoneal cells. *Am. J. Transl. Res.* 10 2590–2599. 30210695 PMC6129511

[B24] Zhang K. Zhang H. Zhou X. Tang W. B. Xiao L. Liu Y. H. (2012). miRNA589 regulates epithelial-mesenchymal transition in human peritoneal mesothelial cells. *J. Biomed. Biotechnol.* 2012:673096. 23118514 10.1155/2012/673096 PMC3479401

[B25] Zhang L. Zhao S. Zhu Y. (2020). Long non-coding RNA growth arrest-specific transcript 5 alleviates renal fibrosis in diabetic nephropathy by downregulating matrix metalloproteinase 9 through recruitment of enhancer of zeste homolog 2. *FASEB J.* 34 2703–2714. 10.1096/fj.201901380rr 31916627

[B26] Zhang S. Cui R. (2018). The targeted regulation of miR-26a on PTEN-PI3K/AKT signaling pathway in myocardial fibrosis after myocardial infarction. *Eur. Rev. Med. Pharmacol. Sci.* 22 523–531. 29424913 10.26355/eurrev_201801_14205

[B27] Zhang X. W. Wang L. Ding H. (2019). Long non-coding RNA AK089579 inhibits epithelial-to-mesenchymal transition of peritoneal mesothelial cells by competitively binding to microRNA-296-3p via DOK2 in peritoneal fibrosis. *FASEB J.* 33 5112–5125. 10.1096/fj.201801111rr 30652956

[B28] Zhao M. Xu P. Liu Z. Zhen Y. Chen Y. Liu Y. (2018). Dual roles of miR-374a by modulated c-Jun respectively targets CCND1-inducing PI3K/AKT signal and PTEN-suppressing Wnt/β-catenin signaling in non-small-cell lung cancer. *Cell Death Dis.* 9:78. 29362431 10.1038/s41419-017-0103-7 PMC5833350

[B29] Zhou J. Zhong J. Lin S. Huang Z. Chen H. Tang S. (2017). Inhibition of PTEN activity aggravates post renal fibrosis in mice with ischemia reperfusion-induced acute kidney injury. *Cell. Physiol. Biochem.* 43 1841–1854. 29049990 10.1159/000484070

